# A physical activity and socioemotional intervention for residents of a large vulnerable community in Brazil during the COVID-19 pandemic: a randomized controlled study

**DOI:** 10.3389/fpubh.2025.1463401

**Published:** 2025-03-14

**Authors:** Mateus Torres-Cruz, Mariana Moura-Alves, Renata Pereira Lima, Rachel King, Cleber Aparecido dos Santos, Thiago da Silva Almeida, Frederico Barão Callamari, Flavia Cristiane Kolchraiber, Márcio Marega, Márcio Henrique Atalla, Edson Amaro, João Ricardo Sato, Elisa Harumi Kozasa

**Affiliations:** ^1^Hospital Israelita Albert Einstein, São Paulo, Brazil; ^2^University of Chichester, Chichester, United Kingdom; ^3^Fred Barão Desenvolvimento Humano, São Paulo, Brazil; ^4^Casa do BemStar, São Paulo, Brazil; ^5^Center for Mathematics, Computing and Cognition, Universidade Federal do ABC, São Bernardo do Campo, Brazil

**Keywords:** emotion regulation, contemplative practices, favela, depression, meditation, exercise, vulnerable populations, COVID-19

## Abstract

**Introduction:**

The COVID-19 pandemic exacerbated mental health issues, particularly in vulnerable communities. Non-psychiatric interventions, including psychological emotional regulation, contemplative practices, and physical activity, can be powerful tools for improving mental health, especially in vulnerable populations. The present study evaluates the effect of a novel low-cost Socioemotional and Physical Activity Intervention in a Brazilian large vulnerable community during the pandemic’s final period.

**Materials and methods:**

Participants were adults (18 to 60 years of age) that resided in the Paraisópolis, the third largest *favela* in Brazil. Recruitment was done through advertising via mobile messaging. Participants were divided into two groups, Intervention (Group I) or Waiting List Control (Group C). Group I participants underwent an in-person Multidimensional Intervention of 1 h per week, for 12 weeks, which was composed of socioemotional skills learning and moderate physical activities, while Group C maintained their usual daily routines. All participants were evaluated before (T0) and after (T1) the Intervention. The evaluation included four validated questionnaires to assess mental health (DASS-21, PANAS, WHO-5 and BRS), IPAQ for evaluating physical activity levels and a physical fitness assessment, which provided quantitative data. A semi-structured interview was also done, which provided qualitative data and was analyzed using a reflexive thematic analysis.

**Results:**

Quantitative data was collected from 88 participants, 43 from Group I and 45 from Group C. We observed a reduction in the scores for depression (DASS−21; Mean difference between evaluations [MD] = −3.2 [± 1.13, SEM], *p* = 0.006) and negative affects (PANAS, MD = −2.7 [± 0.97], *p* = 0.012) observed only in the participants of the I group in T1 compared to T0, but not for the C group. We also found a reduction in systolic arterial blood pressure in hypertensive or pre-hypertensive participants after exercise (Group I *n* = 28; −7.0 [± 2.8] mmHg, *p* = 0.014), an increase in physical endurance (walk test, MD = +56.0 [±8.7] m, *p* < 0.001) and flexibility (sit and reach test, MD = +5.12 [±0.85] cm, *p* < 0.001) only in the I group on T1, compared to T0. The reflexive thematic analysis results suggest that the Intervention not only alleviated negative emotional states, such as anxiety and sadness, but also provided a notable enhancement in participant’s physical vitality, corroborating and complementing the quantitative analysis results.

**Conclusion:**

The results presented here indicate that the Intervention presented here has the potential to reduce symptoms correlated with mental disorders and improve physical fitness in residents of a large vulnerable community.

## 1 Introduction

The COVID-19 pandemic generated a profound impact in the globe, with over 6.8 million deaths worldwide (1) and more than 2.1 trillion of U.S. dollars of economic loss globally (2, 3). The pandemic had a substantial impact on the mental health, increasing the prevalence of depression, anxiety, insomnia and distress (4–11). The pandemic events were more felt in vulnerable communities, particularly in areas of dense population and limited mitigation capacity (12–17). In fact, several countries around the globe implemented a number of different strategies to support these populations by mitigating health and social inequalities (see (18) for a comparative case study of 15 countries). A review found that non-psychiatric mental health interventions can be effective, yet economically accessible, in promoting mental health (19), representing a possible strategy to mitigate inequalities in the context of health-related emergencies.

Non-Psychiatric interventions can take many shapes and forms. Interventions can, for example, promote Socioemotional (SE) regulation strategies to improve mental health (see (20) for a meta-analysis). In this direction, Kemeny et al. found that a training encompassing contemplative practices (such as meditation) and emotion regulation to improve emotional experience and SE behavior reduced negative affect, negative emotional behaviors, and symptoms of depression, anxiety and stress, while promoting prosocial behaviors (21). Unsurprisingly, recent research suggests that contemplative practitioners were better able to cope with social distancing during sanitary emergency period of the COVID-19 pandemic, showing less symptoms correlated with depression (22). Another approach to Interventions is to employ Physical Activity (PA). Evidence shows that PA interventions improve physical fitness, general quality of life, while also preventing and mitigating symptoms of mental health disorders, such as depression and anxiety (see 22 for a review). A recent study shows that during the COVID-19 pandemic, higher physical activity was associated with an age-independent increased well-being, quality of life, and with decreased symptoms of depression, anxiety, and stress (23). It is not clear, however, whether these SE and PA interventions could be effective in vulnerable populations.

In the current scientific literature, most interventions are assessed in high-income geographic areas, even if they involve low-income populations. Although vulnerable populations are less likely to engage in health promoting activities due to lack of opportunities and more pressing life-threatening conditions, e.g., nutrition insecurity, strategies such as tailoring, providing social support and using a multidisciplinary approach are promising toward providing them effective Interventions (24). In this scenario it is key to investigate the effects of health interventions in low-income populations—in particular, multidimensional strategies adapted to their specific needs and vulnerabilities. Therefore, our goal in this study was to evaluate the effects of a novel low-cost Multidimensional Intervention, which combines PA and SE learning, on mental and physical health outcomes a vulnerable population.

This study was performed in Paraisópolis, the third largest *favela* of Brazil. “*Favela*” is a Brazilian Portuguese umbrella term to refer to slums, ghettos, shantytowns, or squatter settlements, characterized by densely populated urban areas with inadequate infrastructure and limited access to essential services, on which some vulnerable populations of Brazil live. There are approximately 58,527 inhabitants in Paraisópolis (25, 26) and the average income per family is estimated between 170 and 560 USD^1^ (27, 28). Besides grappling with challenges such as unemployment, informal labor, and a precarious healthcare access, research shows that this *favela,* similar to other population groups, was severely affected by the COVID-19 pandemic (29). Thus, we argue it is a representative place to investigate the effect of interventions on vulnerable communities.

The Intervention consisted of an original in-person 12-week program, with one 1-h class per week, each composed of 40 min of moderate PA and 20 min of contemplative practices coupled with SE learning. In-person practices were complemented by videos with additional PA exercises and meditation audio guides sent each week. The practices were designed to be relevant for their reality, for example, with PA exercises using everyday household items instead of specialized equipment, SE learning component based on their daily experiences and contemplative practices being short and easy-to-follow. They were also designed to facilitate the training of non-specialists in mental health to deliver it, making it viable as a strategy to promote mental health in times of scarcity of mental health professionals (19).

Based on the results of aforementioned studies, our hypothesis was that the intervention would reduce negative moods, emotions and behaviors, decrease symptoms of depression, anxiety and stress, and increase physical fitness of the participants. To test that, we employed a randomized controlled design, such that all participants were evaluated before and after the intervention and the design allowed comparing the results from the group receiving the intervention to a waiting list control group that did not receive the intervention. We employed several validated questionnaires, such as the Depression, Anxiety and Stress Scale (DASS-21), to evaluate mental health and performed a physical fitness assessment, including tests of flexibility, cardiac function and others, to evaluate physical health. Our results indicates that our Multidimensional Intervention reduced symptoms of depression and the occurrence of negative moods or emotions, while also increasing the participants physical endurance and flexibility.

## 2 Materials and methods

The study design was a randomized controlled study. The study was approved by the Research Ethics Committee of Hospital Israelita Albert Einstein, under national research registration number CAAE 52307821.7.0000.0071.

### 2.1 Participants

Participants were adults living in the Paraisópolis favela, in the city of São Paulo, Brazil. The inclusion criteria comprised of (I) living in Paraisópolis at the time of expressing interest in participating in the study and (II) having a schedule compatible with participating in the Intervention’s weekly activities (see below). The exclusion criteria comprised of (I) having less than 18 or more than 60 years of age, (II) taking medicine for any psychiatric disorder (self-report), (III) having any physical condition that would prevent the participant from performing the physical activities required by the intervention (either self-reported or identified by the study’s physical educators) and (IV) having an active COVID-19 infection (self-report).

The study was conducted with the help of the *Programa Einstein na Comunidade de Paraisópolis* (PECP). The PECP is a Social Responsibility initiative from the “Albert Einstein Brazilian Israelite Beneficent Society” (free translation of “*Sociedade Beneficente Israelita Brasileira Albert Einstein*”) which offers, free of charge, several social-educational activities, working within six axes: Art and Communication, Professional Training, Education, Sports, Health, and Social Work. They have a 5,500 square meters complex built in the heart of the *favela,* have been a part of the Paraisópolis community since 1998 and, as of 2021, served over 5,000 people per year (30).

We used PECP’s contact network to advertise the study. By July of 2022 this network consisted of several WhatsApp broadcast lists and groups, which reach out directly to an estimate of one to 2,000 people in the community; it is known, however, that people in the community usually forward this kind of ad, so many more people were indirectly reached. This network is used by PECP as its main media vehicle to broadcast their courses, campaigns, and initiatives. Although there are inequalities in internet access and smartphone ownership in Paraisópolis compared to the rest of the country (31), we chose to use WhatsApp because it is the most used messaging app in Brazil, with 96% of smartphone users in the country being an active in the platform (32), and more than 91% of Paraisópolis’ inhabitants own a smartphone (33). Also most major phone carriers in the country—including “Paraisópolis Celular,” a carrier based on the community—do not count data usage on WhatsApp toward total data usage in mobile plans (31). The promotional material consisted of a series of digital flyers which included the following text:

“Do you want to improve your physical and mental health? Participate in our free practices facilitated by physical educators and psychologists! Includes exclusive videos by Marcio Atalla^2^. On PECP from Aug/2022 on. Registration at: <link to a redcap survey>”.

A minor part of the participants was approached directly by one of the investigators (MTC) when they were in the PECP facilities to participate in some other activity. In both cases, the only information participants had access at enrollment was that they were registering for “free activities aimed at promoting their physical and mental health.”

### 2.2 Groups and schedule

Approximately 3 weeks after the beginning of the registration period (August 2022), selected participants were invited to take part in the initial evaluation (T0). This evaluation occurred over five separate days within a two-week timeframe, allowing for flexibility to accommodate various participant schedules.

On the day of the initial evaluation, following an elucidation of the study’s phases and the completion of the Research Consent Form, participants engaged in responding to a set of validated questionnaires and underwent physical evaluation. Detailed descriptions of these procedures can be found in the sections below. Subsequently, participants were randomly assigned to either the Intervention (I) or Waiting List Control (C) groups using the RAND() function of the online version of Microsoft Excel 365 (34).

Group I initiated the 12-week intervention immediately following the T0 evaluation, while Group C maintained their usual daily routines. At the conclusion of the 12-week intervention period, all participants, including those in Group C, underwent a second evaluation (T1) identical to T0. Group C participants were offered the same intervention as Group I after the completion of T1.

### 2.3 The intervention

The Intervention was constituted of an original 12-week program consisting of two distinct parts: Physical Activity and Socioemotional Learning. The physical activity portion was based on functional fitness training principles, while the Socioemotional learning was based on the Cultivating Emotional Balance training program, which associates contemplative practices with techniques to improve emotional self-awareness and regulation (21); a detailed description of both is provided below. The practices were designed to be relevant for their reality, for example, with PA exercises using everyday household items instead of specialized equipment, SE learning component based on their daily experiences and contemplative practices being short and easy-to-follow. Participants were required to attend a weekly 1-h presential session. Each session had 40 min of physical activity followed by 20 min of socioemotional learning. In addition to the presential sessions, at least one 2 min-long physical activities video (with exercises both novel and practiced during the sessions) and 10 min-long contemplative practice audio-guide (the same practice done during the session) were sent to the participants after each session via WhatsApp. Participants that were not on intervention period received a weekly video with general Health Education content.

Participants did not receive monetary compensation for participating in the study, they were, however, refunded for the values they spent on mobile cell data plans (due to the videos that were sent) and transportation, up to 9.35 USD^3^ per month, for 6 months; further, a snack^4^ was offered after each session.

#### 2.3.1 Physical activity

The physical activity part included low impact and intensity exercises, which did not require specialized equipment, used the body as overload, worked out muscle groups in the whole body and focused on improving general mobility and core strength. The exercise routines were designed to be easy to be practiced at home, hence required little space (usually 1 m^2^ or less) and sometimes used common household items (such as chairs or brooms).

The general schedule of the sessions throughout the weeks was as follows:

- Week 1: Core Training and Contraction Techniques. Teaching the nomenclature of muscles and forms of contraction.
- Week 2: Breathing Techniques. How to breathe during exercises? Explanation of the importance of using the correct breathing technique and its benefits through exercises.
- Week 3: Joint movements and exercises using a broomstick. Exercises for the upper and lower limbs using broomsticks and wooden poles.
- Week 4: Coordination and Rhythm—Part 1. Activities with mini hula hoops placed on the floor and coordination exercises in and out of them, associated with upper limb movements.
- Week 5: Coordination and Rhythm Exercises—Part 2. Activities with mini hula hoops placed on the floor and coordination exercises, getting in and out them, associated with upper limb movements. This class used more complex changes of direction and greater speed compared to the previous.
- Week 6: How to squat? Explanation of postures, angles, and types of squats. Performance of some types of squats.
- Week 7: What exercises can be done using tension bands? Explanation of the distinct levels of tension of elastic bands and practice of exercises for upper and lower limbs using them.
- Week 8: Exercises with a tennis ball. Activities to explore a tennis ball, in terms of weight, size and movement possibilities, followed by muscle and coordination exercises using a ball.
- Week 9: How can I unify my workouts? Explanation of how circuit training works, guidance, and demonstration of the possibilities for training variations of exercises learned in previous weeks. Circuit training using exercises from previous classes.
- Week 10: Laterality and dissociation. Explanation of what laterality is and the importance of being able to dissociate movements between sides and lower and upper limbs. Promotion of activities that encourage dissociation between limbs and sides.
- Week 11: Core stabilization in rotations. Questions about the topic were answered, the importance and functions of the core rotator muscles were explained, and core stabilization exercises were done in pairs and with balls.
- Week 12: Possibility of exercises with fabrics (towels, vests, and cloths). Demonstration of how to use fabric for exercises and practice of lower and upper limb exercises using fabric.

All the classes began with joint warm-ups and specific stretches for the muscles being worked on. The number of sets, repetitions and use of variations was adapted based on the fitness levels of the participants present in each session. Participants that showed above average or below average fitness levels were encouraged and instructed to increase or decrease exercise intensity in relation to the rest of the participants. The 2-min physical activity video that was sent out after each session showed variations of that day’s exercises.

A more thorough description of exercises can be found in the Supplementary material.

#### 2.3.2 Socioemotional learning

The Socioemotional Learning part was based on the Cultivating Emotional Balance (CEB) training, which associates modern psychology, current emotion research and contemplative practices to improve emotional self-awareness and regulation (21). CEB training was conducted by certified instructors of the program. Every session began with a very brief recapitulation of what was discussed in the previous session and a moment in which participants could share how they applied what they have learned in their daily lives since then. A brief explanation of the current week’s theme and related concepts was given by the instructor, accompanied by a 10-min-long contemplative practice.

The schedule for the Socioemotional Learning part of the presential sessions throughout the weeks was as follows:

- Week 1: What are emotions and what are their consequences on our actions?
  A brief introduction to meditation. Contemplative practice: Relaxation of the body and mind.
- Week 2: Why do we think what we think and feel what we feel? Contemplative practice: Relaxation of the body and mind.
- Week 3: The timeline of an emotional episode. Contemplative practice: Focusing the mind on the abdomen.
- Week 4: Anger and how to deal with it. Contemplative practice: Focusing the mind on the abdomen.
- Week 5: Joy and its importance in our lives. Contemplative practice: Meditation for nurturing gratitude.
- Week 6: Fear: what it is and its triggers. Contemplative practice: Meditation for nurturing loving kindness and compassion.
- Week 7: Hedonic and Eudaimonic Happiness. Contemplative practice: Meditation for nurturing loving kindness and compassion.
- Week 8: Anxiety and its relation to fear. Contemplative practice: Deep breathing and natural breathing while focusing the mind on the abdomen.
- Week 9: Sadness and its many types. Contemplative practice: Meditation for nurturing compassion.
- Week 10: How to focus on what is good in life and savor every moment. Contemplative practice: Meditation for nurturing empathetic joy.
- Week 11: In life, there are good days and bad days—how to deal with them? Contemplative practice: Meditation for nurturing empathetic joy.
- Week 12: Recap of the Intervention and prospects for continuing to apply what was learned. Contemplative practice: Meditation for nurturing gratitude and celebration of oneself.

Ten minutes-long audio guides were sent after each session and had the same contemplative practice as the one performed during that same day’s session.

The lesson plans and meditation text scripts can be found in the Supplementary material.

### 2.4 Quantitative data collection and analysis

Study data were collected and managed using REDCap (Research Electronic Data Capture) web-based software, hosted at Hospital Albert Einstein (35, 36). Participants received an ID to be anonymized during the analysis and in accordance with the Brazilian General Data Protection Law (37).

Data collection captured two moments of the intervention: T0 and T1. As explained in “Groups and schedule” section, T0 was a pre-intervention assessment and T1 a post-intervention assessment for Group I, while Group C participants continued their regular daily routines throughout assessments.

#### 2.4.1 Instruments

The data collection instruments employed were the following. Every instrument was present in all evaluations unless specified otherwise.

- Personal data form. Included information such as name, date of birth, contact information and personal identification document numbers. It was only present in full at T0; contact information was updated in posterior collection events.
- Health information form. Identified preexisting conditions, recent diseases, or drugs currently in use that could fit in the exclusion criteria.
- IPAQ—International Physical Activity Questionnaire: short form. Measures levels of regular physical activity (PA). It calculates scores for three types of activities: walking, moderate PA, and vigorous PA. These scores are reported as MET-minutes of each activity per week (METs are multiples of the resting metabolic rate). The MET scores used in this study were: walking = 3.3 METs, moderate PA = 4.0 METs and vigorous PA = 8.0 METs. Total scores (sum of the three types) range from 0.0 to 53,760.0. IPAQ has a test–retest validity of 0.75 and a reliability of 0.71 in the Brazilian population (38, 39).
- DASS-21—Depression, Anxiety and Stress Scale. Measures levels of three components related to different emotional states: depression, anxiety, and stress. Each component has seven questions which use a four-point Likert-type scale, resulting in a specific score for each component and a total score indicating general psychological distress. Scores were multiplied by two to make results comparable to the complete scale (DASS-42) and range from 0 to 168, with higher values indicating more distress. Internal consistency, as measured by Cronbach’s alpha is 0.92 for the depression, 0.90 for the stress, and 0.86 for the anxiety components (40, 41).
- WHO-5—World Health Organization Well-Being Index. Measures the level of self-reported subjective mental well-being. It comprises five 5-point Likert-type scale questions, which scores are summed and multiplied by four to generate a percentage, which ranges from 0 to 100, with higher values indicating more well-being. Internal consistency as measured by Cronbach’s alpha is 0.83 (42).
- PANAS—Positive and Negative Affects Schedule. Includes one scale to measure positive affects (moods or emotions), with nine items [on its version in Brazilian Portuguese (43)], and one to measure negative affects, with 10 items. Each item is a five-point Likert-type question. The total score for each scale is calculated separately by summing all items in the scale, which ranges from 0 to 45 on the positive scale and from 0 to 50 in the negative scale, with higher values indicating more intense affects. The negative and positive scales show a Cronbach’s alpha of 0.87 and 0.88, respectively (43, 44).
- BRS—Brief Resilience Scale. A five-item scale [on its version in Brazilian Portuguese (45)] that assesses one’s perceived ability to “bounce back” or recover after a stressful event (45, 46). Each item is a five-point Likert-type question. Three items are positively worded and three are negatively worded. The final score is obtained by reverse coding the negatively worded items and finding the mean of the six scores (range 0–25). The test has consistency as measured by Cronbach’s alpha of 0.76 (45).
- Physical assessment. It constituted several measurements and tests aimed at obtaining anthropometric and cardiovascular measurements and measuring the general fitness of the participants. It included the following: body mass index (BMI), waist-to-height ratio, flexibility on the Sit and Reach Test and the max number of sit-ups the participant can do in 1 min. Additionally, a 6 min’ walk test was performed, on which the maximum distance the participant could walk within 6 min was calculated. The test was done in 20 meters-long linear non-inclined asphalt-coated circuit on which the subject could lap freely. The number of complete laps multiplied by the size of the circuit summed to the number of meters walked in the last incomplete lap was used to calculate the number of meters walked. The walk test was preceded and followed by a measurement of the participant’s arterial blood pressures and cardiac frequency, such that pre and post physical exercise cardiovascular performance could be assessed.
- Semi-structured qualitative assessment. The assessment used a Collective Interview, which can portray the experiences lived by people, groups or organizations and facilitate a detailed understanding of beliefs, feelings, attitudes, and values; here it was used to get to know individual perceptions about the Intervention project and its meaning in their lives. It was only present in T1. The following questions were used as starting point:

o What is your perception of practicing what you learned in the intervention in your daily life?
o How do you feel physically about practicing what you learned in the Intervention?
o How do you feel mentally and emotionally about practicing what you learned in the Intervention?
o How are your relationships after the practice of what you learned in the Intervention?

#### 2.4.2 Analysis

Quantitative data analysis was performed using R version 4.2.3 (47) in the RStudio integrated development environment version 2023.6.1.524 (48). Data import, tidying, manipulation, visualization, and programming was primarily done using the “tidyverse” package version 2.0.0 (49), with the aid of “readxl” 1.4.2 (50), “broom” 1.0.4 (51) and “cowplot” 1.1.1 (52). The package “lme4” 1.1.32 (53) was used to fit models and “lmerTest” 3.1.3 (54) to obtain *p*-values returned by them. Estimated marginal means and contrast tests were obtained using “emmeans” 1.8.5 (55). The “knitr” 1.42 (56–58) and “kableExtra” 1.3.4 (59) were used for internal report generation.

The analysis of the results included the following dependent variables: DASS-21 anxiety, depression, stress, and total score; PANAS negative affect and positive affect scores; WHO-5 well-being index; BRS resilience score; IPAQ physical activity level scores (METs) for walking, moderate PA, vigorous PA, and total score; systolic and diastolic blood pressure, and heart rate, taken before and after the walk test; the number of meters walked in the walk test; flexibility in cm in the sit and reach test; maximum number of sit-ups performed in 1 min; BMI; and waist-to-height ratio. Each of the variables was analyzed separately.

The variables whose distribution showed significant deviations from the normal distribution were analyzed with a non-parametric approach and the rest with a parametric approach. To decide which approach would be used for each dependent variable (DV), a separate Shapiro–Wilk’s test was run, and a Q-Q plot was drawn, for each DV. DVs that had a significant test and/or showed important deviations of the normal distribution were analyzed using the non-parametric approach; the remaining were analyzed using the parametric approach. If the model diagnostics for a DV being analyzed using the parametric approach indicated poor fit, the DV was analyzed using the non-parametric approach. The parametric approach used ANOVA with group (I and C) as the between-subjects factor and assessment (T0 and T1) as the within-subjects factor, which was followed by a multiple comparison test with Bonferroni correction when necessary (60–62). The non-parametric approach comprised of Wilcoxon tests to compare T0 and T1 within groups, followed by Mann–Whitney tests on the delta (difference between T1 and T0) between the groups (63); in this case, differences between groups were only considered when both Wilcoxon and Mann–Whitney results reached the statistical threshold.

All quantitative analysis considered *p*-values <0.05 to be statistically significant.

### 2.5 Reflexive thematic analysis

At T1, participants that had already gone through the intervention were invited to participate in the semi-structured qualitative assessment in the form of a collective interview (see above). Participants were recruited in groups of 3 to 5 people through convenience sampling. Individuals expressed their availability on the last day of data collection.

Interviews were conducted in person, audio-recorded using a Samsung A32 mobile phone and then transcribed and translated by a member of the research team. Interview transcripts were anonymized and imported into NVIVO12 (64).

The thematic analysis was done according to the six-phases of Braun and Clarke’s reflexive thematic analysis method: (1) Familiarization of data, (2) Generation of codes, (3) Combining codes into themes, (4) Reviewing themes, (5) Determine significance of themes and (6) Reporting of findings (65).

## 3 Quantitative results

**Figure 1:**
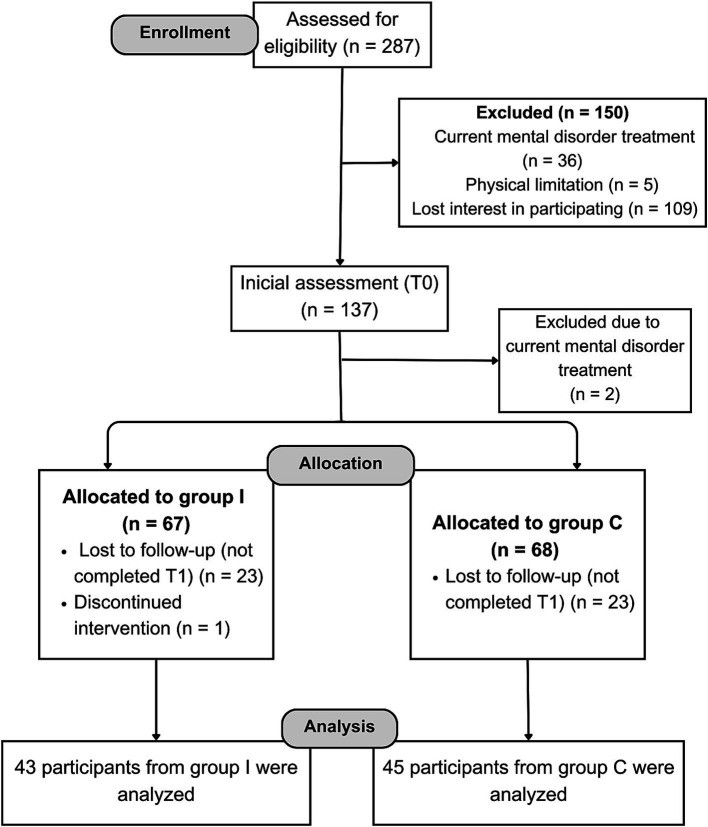
Flowchart of recruitment of participants.

Data analysis included data from 88 participants, 43 from Group I and 45 from Group C. A flowchart describing more details about participants can be found below (see Figure 1). Initially, 67 participants were allocated to group I; 1 participant was asked to discontinue the intervention because we found out after the beginning of the classes that they did not have 18 years of age, and the remaining 23 participants were lost to follow-up. Of those 23, 12 withdrew their participation because they no longer had time to participate, either due to starting a job (*n* = 3), changes in their schedule (*n* = 6), emergency travel (*n* = 1), feeling unable to follow the PA exercises (*n* = 1) or lack of interest (*n* = 1); and 11 were excluded from the analysis due to having less than 75% of attendance. Therefore, all 43 of the included Group I participants had 75% or more of attendance (mean = 93%, median = 92%, min = 75%, max = 100%).

Although the activities were widely publicized in the community, most participants were women (85 participants, 96.59%), which was common for activities promoted by the PECP.

The analysis reported below compares the T1 and T0 assessments, therefore the reported values refer to the delta, i.e., the mathematical result from “T1-T0” operation, thus a positive score indicates T1 > T0, or an increase in the score throughout assessments, in the format “mean [±standard deviation]” for variables analyzed using a parametric approach and in “median [interquartile range expressed as ‘1^st^ quartile to 3^rd^ quartile’]” when the non-parametric approach was used. Table 1 contains the results for the variables analyzed using the non-parametric approach and Table 2 contains the results for the variables analyzed using the parametric approach.

**Table 1:** Within-group T0 and T1 Wilcoxon analysis followed by between-group Mann–Whitney tests on the delta (difference between T0 and T1, i.e., T1–T0).

Dependent variable	Scores								Deltas (T1-T0)			
									Median (IQR)		Mann–Whitney	
	Median (IQR)		Wilcoxon		Median (IQR)		Wilcoxon		Intervention	Waiting list control	U	*p*
	T0	T1	V	*p*	T0	T0	V	*p*				
DASS Total	16 (6 to 30)	14 (5 to 22)	550	**0.061**	12 (4 to 22)	10 (2 to 22)	294	0.823	-2 (−15 to 5)	0 (−6 to 2)	838	0.280
**DASS Depression**	6 (2 to 13)	4 (0 to 10)	456	**0.007**	4 (0 to 8)	4 (0 to 8)	204	0.552	-2 (−7 to 0)	0 (−2 to 2)	644	**0.006**
DASS Anxiety	2 (0 to 6)	2 (0 to 4)	290	0.627	2 (0 to 6)	2 (0 to 4)	184	0.559	0 (−2 to 2)	0 (−2 to 0)	998	0.800
DASS Stress	8 (2 to 13)	8 (2 to 10)	380	0.292	4 (0 to 12)	4 (0 to 10)	192	0.810	0 (−6 to 3)	0 (−2 to 2)	896	0.548
PANAS Positive affect	29 (21 to 32.5)	30 (26 to 35)	255	**0.038**	31 (22 to 34)	29 (25 to 36)	366	0.558	3 (−2.5 to 5)	0 (−3 to 4)	1,100	0.270
**PANAS Negative affect**	22 (18.5 to 26.5)	20 (16 to 24)	626	**0.012**	19 (15 to 25)	20 (17 to 26)	382	0.384	−3 (−7.5 to 2)	1 (−4 to 7)	674	**0.014**
WHO-5	52 (36 to 76)	68 (34 to 80)	304	0.155	56 (32 to 76)	60 (40 to 80)	378	0.499	4 (−6 to 20)	4 (−12 to 12)	1,036	0.572
IPAQ Total	1022.5 (305.3 to 3710.3)	1911 (976.1 to 3487.5)	304	**0.066**	895 (396 to 1,695)	1836 (412.5 to 4,068)	300	**0.023**	963 (−437.3 to 2,100)	495 (−455 to 1,668)	960	0.902
IPAQ vigorous PA	0 (0 to 0)	480 (0 to 1,440)	135.5	**0.077**	0 (0 to 0)	0 (0 to 1920)	55.5	**0.012**	480 (0 to 960)	0 (0 to 720)	1,017	0.529
IPAQ moderate PA	0 (0 to 1,050)	400 (240 to 1,140)	333	0.302	0 (0 to 480)	240 (0 to 1,280)	140	**0.098**	240 (−330 to 525)	0 (0 to 960)	967	0.854
IPAQ walking	396 (0 to 767.3)	544.5 (210.375 to 990)	256	**0.063**	495 (198 to 660)	412.5 (198 to 990)	436	0.856	198 (−24.7 to 445.5)	0 (−297 to 396)	1,096	0.201
Pre-test SBP	130 (121 to 145.5)	126 (114 to 138.5)	596	**0.032**	121 (116 to 129)	118 (111 to 129)	586	0.172	-4 (−10.5 to 2)	-3 (−8 to 5)	900	0.576
Post-test SBP	130 (116 to 140.5)	126 (117.5 to 135)	476	0.378	119 (110 to 128)	117 (108 to 125)	608	0.312	-3 (−10 to 8.5)	−2 (−8 to 6)	957	0.933
Pre-test DBP	84 (79 to 96)	85 (77 to 91.5)	412	0.989	80 (74 to 85)	80 (75 to 88)	431	0.616	1 (−4 to 3)	1 (−4 to 5)	904	0.598
Post-test DBP	85 (77.5 to 99)	86 (78.5 to 93.5)	504	0.717	81 (75 to 87)	80 (75 to 87)	428	0.769	1 (−5 to 3.5)	0 (−5 to 5)	926	0.735
^§^Pre-test SBP	142 (130.7 to 152)	132 (124 to 148.7)	289	**0.051**	135 (130 to 138.5)	128 (119 to 133)	77.5	0.124	−5 (−16.7 to 3.7)	−4 (−13.5 to 2)	212	0.980
^§^Post-test SBP	136.5 (130.7 to 154.5)	130.5 (123 to 143.7)	272	**0.014**	130 (120 to 138)	130 (117.5 to 135.5)	56	0.842	−6 (−12.3 to 0.250)	−2 (−4.5 to 6.5)	132	0.050
^§^Pre-test DBP	92 (84.7 to 101.3)	87.5 (83.7 to 96)	215.5	0.315	89 (86 to 93.5)	90 (84 to 94)	67.5	0.690	−2 (−6.5 to 3.25)	1 (−4 to 3)	197	0.750
^§^Post-test DBP	95.5 (84.7 to 101.3)	90 (85.5 to 98.7)	251.5	0.274	92 (84 to 94)	92 (85.5 to 94.5)	50.5	0.925	−2 (−6 to 3.25)	0 (−4.5 to 5)	188	0.574
**Pre-test HR**	74 (65.5 to 81.5)	76 (67.5 to 83)	396	0.487	75 (68 to 80)	80 (73 to 87)	190	**<0.001**	1 (−5 to 7.5)	6 (−1 to 12)	707	**0.030**
Post-test HR	75 (70 to 85)	80 (73 to 89.5)	323	**0.071**	80 (71 to 86)	84 (76 to 93)	176	**0.001**	5 (−2.5 to 9.5)	6 (−1 to 11)	904	0.599
^§^Pre-test HR	76 (70 to 81.3)	78 (70.3 to 86.5)	177	0.561	81 (75.5 to 91)	89 (81 to 98)	35.5	0.172	0 (−5 to 8)	6 (−4.5 to 11)	180	0.444
^§^Post-test HR	78 (71.5 to 89.5)	82.5 (76 to 89.7)	112	**0.039**	86 (80 to 101)	95 (85.5 to 99.5)	22	0.195	6 (−2 to 9.25)	0 (−3 to 10.5)	226	0.693
Sit-ups	8 (0 to 15)	13 (1 to 19)	38	**<0.001**	14 (0 to 19)	16 (0 to 21)	68.5	**0.004**	2 (0 to 5)	0 (0 to 5)	1,127	0.176
Body mass index	27.8 (24.9 to 32.7)	28.3 (25.5 to 33.4)	156	**<0.001**	29.1 (24.5 to 33.5)	29.5 (24.8 to 33.9)	198	**<0.001**	0.38 (0.095 to 0.840)	0.47 (0.090 to 1.07)	929	0.751
^#^Body mass index	31.2 (27.5 to 33.3)	31.9 (27.7 to 34.2)	79	**0.001**	31.6 (28.5 to 34)	32.3 (29.3 to 34.7)	97	**0.002**	0.395 (0.123 to 0.775)	0.575 (−0.170 to 0.973)	491	0.785

Differences between groups were only considered when both Wilcoxon and Mann–Whitney results were statistically significant (*p* < 0.050); instances where this happened are noted with a bold and underlined Dependent Variable. *p* values between 0.100 and 0.050 are emphasized in bold font, whereas *p* values <0.050 are emphasized in bold and underlined font. *p* values smaller than 0.001 were noted as “*p* < 0.001.” Data analysis included data from 88 participants, 43 from Group I and 45 from Group C. “§” indicates analysis on which only participants with prehypertension and hypertension were included (*n* Intervention [I] = 28; *n* Waiting List Control [C] = 15). “#” indicates analysis on which only overweight and obese participants were included (*n* I = 32; *n* C = 32). DASS: Depression, Anxiety and Stress Scale—21 items. PANAS: Positive and Negative Affects Schedule. IPAQ, International Physical Activity Questionnaire—short form. PA, Physical Activity. Pre-test and Post-test refers to the 6 min’ walk test. SBP, Systolic Blood Pressure. DBP, Diastolic Blood Pressure. HR, Heart Rate.

**Table 2:** Statistical analysis results for variables analyzed using ANOVA.

Dependent variable	Scores mean (SD)				Deltas (T1-T0) mean (SD)		ANOVA					
	Intervention		Waiting list control		Intervention	Waiting list control	Group		Assessment		Group*assessment	
	T0	T1	T0	T1			F(df)	*p*	F(df)	*p*	F(df)	*p*
BRS	3.12 (0.773)	3.23 (0.672)	3.39 (0.783)	3.35 (0.756)	0.112 (0.732)	−0.036 (0.775)	2.018 (1;86)	0.159	0.205 (1; 86)	0.652	0.837 (1;86)	0.363
**Walk test**	464 (73.1)	519 (51.0)	490 (48.2)	456 (42.3)	54.6 (57.3)	−33.8 (50.1)	3.22 (1;86)	**0.076**	2.67 (1;86)	0.106	59.60 (1;86)	**<0.001**
**Flexibility**	22.3 (10.6)	27.4 (10.7)	26.1 (10.9)	28.0 (11.4)	5.12 (5.60)	1.82 (7.48)	0.947 (1;86)	0.333	23.598 (1;86)	**<0.001**	5.433 (1;86)	**0.022**
Waist-to-height ratio	0.579 (0.087)	0.575 (0.083)	0.561 (0.097)	0.569 (0.089)	−0.003 (0.023)	0.007 (0.035)	0.40 (1;86)	0.529	0.39 (1;86)	0.534	2.70 (1;86)	0.104
^#^Waist-to-height ratio	0.613 (0.072)	0.606 (0.071)	0.601 (0.085)	0.606 (0.076)	−0.007 (0.025)	0.005 (0.040)	0.088 (1;62)	0.767	0.058 (1;62)	0.811	1.973 (1;62)	0.165

Group (I and C) was used as the between-subjects factor and assessment (T0 and T1) as the within-subjects factor. Dependent variables with statistically significant results are noted with bold and underlined fonts. BRS: Brief Resilience Scale. “#” indicates analysis on which only overweight and obese participants were included (n I = 32; n C = 32). See Table 1 for the remaining conventions.

### 3.1 DASS-21—depression, anxiety, and stress scale

A total score for DASS-21 was calculated and subjected to non-parametric analysis along with a separate analysis for the scores of the three components of this scale.

**Figure 2:**
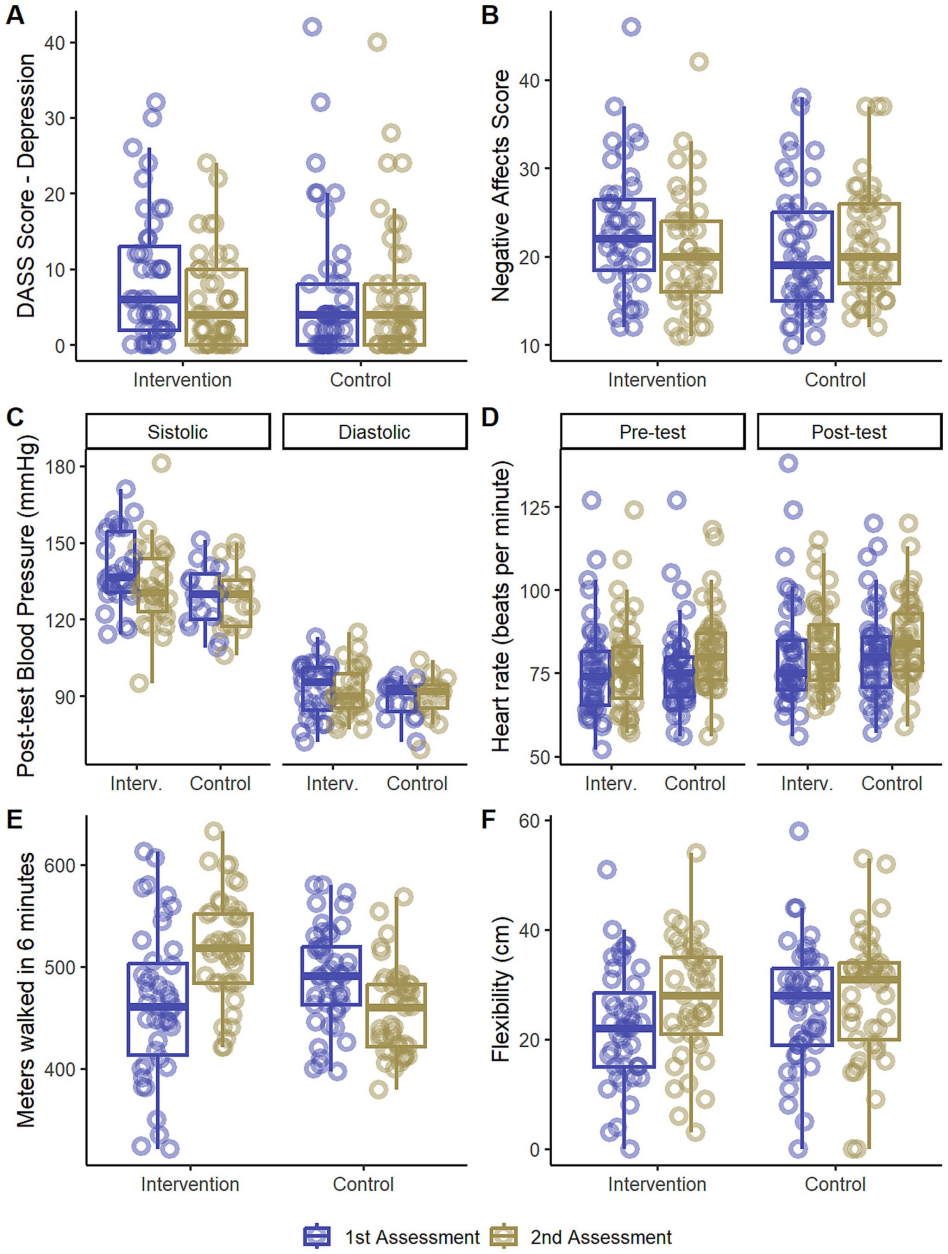
Box plots of statistically significant quantitative analysis. **(A)** DASS-21 scores for the depression component, **(B)** negative affect scores from the PANAS (Positive and Negative Affect Scale), **(C)** blood pressure by type (systolic or diastolic) after the walk test, **(D)** heart rate by moment (before or after the walk test), **(E)** 6-min walk test and **(F)** sit and reach test. All plots are divided by group (x axis, Waiting List Control Group [Control], and Intervention) and assessment (blue indicates first assessment [T0], yellow indicates second assessment [T1]). For panels **(A,B,C,D)** “*” represents a significant difference (*p* < 0.05) the Wilcoxon test comparing assessments AND a significant result in the Mann–Whitney test comparing the difference between assessment 1 (T0) and 2 (T1; score at T1—score at T0) between the groups. For panels **(E,F)** “*” represent a difference in the multiple comparison test with Bonferroni correction in the analysis after a significant Assessment*Group interaction. The Intervention group was made up of 43 participants and the Waiting List group was made up of 45. The overlapping open circles indicate individual data. The thick horizontal line in the center of the boxes represents the median. The bottom of the box is the first quartile (Q1), and the top of the box is the third quartile (Q3). The vertical lines represent the lower and upper limits of the data (Q1–1.5 * Range between quartiles and Q3 + 1.5 * Range between quartiles, respectively).

The depression component score variation between T1 and T0 differed between groups (Mann–Whitney *p* = 0.006), indicated that while Group I presents a reduction in scores between T0 and T1 (median delta [1^st^ quartile to 3^rd^ quartile] = −2 [−7 to 0] points, Wilcoxon *p* = 0.007), in Group C the score remains stable (0 [−2 to 2] points, Wilcoxon *p* = 0.552), as can be seen in Figure 2A. This suggests that the intervention promotes a decrease in the prevalence of depression symptoms.

No differences were found between groups in the anxiety (Group I: 0 [−2 to 2], Group C: 0 [−2 to 0], Mann–Whitney *p* = 0.800), stress (Group I: 0 [−6 to 3], Group C: 0 [−2 to 2], Mann–Whitney *p* = 0.548), and total scores (Group I: 2 [−15 to 5] points, Group C: 2 [−15 to 5] points, Mann–Whitney *p* = 0.280; data not shown).

### 3.2 PANAS—positive and negative affect scale

The Positive Affect and Negative Affect scales were analyzed separately.

A reduction in the Negative Affect score was found in Group I after the intervention −3 [−7.5 to 2] points, Wilcoxon *p* = 0.012, Mann–Whitney *p* = 0.014, but not in Group C (1 [−4 to 7] points, Wilcoxon *p* = 0.384; see Figure 2B). This suggests that the intervention decreases the prevalence of negative moods and emotions.

No differences were found between groups in the scores involving Positive Affects (Group I: 3 [−2.5 to 5], Group C: 0 [−3 to 4], Mann–Whitney *p* = 0.270; data not shown).

### 3.3 WHO-5—well-being index of the world health organization

No differences were found between groups in the results involving the WHO-5 (Group I: 4 [−6 to 20], Group C: 4 [−12 to 12], Mann–Whitney *p* = 0.572; data not shown).

### 3.4 BRS—brief resilience scale

The ANOVA for the BRS scores did not show statistically significant effects [Group: F(1; 86) = 2.02, *p* = 0.159; Assessment: F(1; 86) = 0.21, *p* = 0.652; Group*Assessment: F(1; 86) = 0.84, *p* = 0.363; data not shown].

### 3.5 IPAQ—international physical activity questionnaire

No differences were found between groups in the results involving neither the total IPAQ score (Group I: 963 [−437.3 to 2,100] MET.min^−1^, Group C: 495 [−455 to 1,668], Mann–Whitney *p* = 0.902), nor the vigorous PA (Group I: 480 [0 to 960], Group C: 0 [0 to 720], Mann–Whitney *p* = 0.529), moderate PA (Group I: 240 [−330 to 525], Group C: 0 [0 to 960], Mann–Whitney *p* = 0.854) or walking scores (Group I: 198 [−24.7 to 445.5], Group C: 0 [−297 to 396], Mann–Whitney *p* = 0.201; data not shown).

### 3.6 Cardiovascular variables

Systolic Blood Pressure (SBP), Diastolic Blood Pressure (DBP) and Heart Rate were measured before and after the walk test, resulting in 6 variables, which were analyzed separately.

When all participants were included in the analysis, no significant differences were observed between T0 and T1 in SBP, neither before (Group I: −4 [−10.5 to 2] mmHg; Group C: −3 [−8 to 5]; Mann- Whitney *p* = 0.576), or after the walk test (Group I: −3 [−10 to 8.5]; Group C: −2 [−8 to 6]; *p* = 0.933). The same also applies to DBP pre- (Group I: 1 [−4 to 3]; Group C: 1 [−4 to 5]; *p* = 0.598) and post-test (Group I: 1 [−5 to 3.5]; Group C: 0 [−5 to 5]; *p* = 0.685; data not shown).

However, after seeing these results we hypothesized that changes in SBP and DBP would only be observed in the subset of individuals with hypertension or prehypertension, that is, with SBP greater than 129 mmHg and/or DBP greater than 84 mmHg at rest in the T0 assessment (66). This subgroup had a total of 28 participants in Group I and 15 participants in Group C.

Indeed, in the analysis involving only prehypertensive and hypertensive individuals, a reduction in SBP after the walk test in Group I was found (−6 [−12.3 to 0.250] mmHg, Wilcoxon *p* = 0.014, Mann–Whitney *p* = 0.050), but not in Group C (−2 [−4.5 to 6.5], Wilcoxon *p* = 0.842; see Figure 2C). There were no effects on SBP measured before the walk test (Group I: −5 [−16.7 to 3.7]; Group C: −4 [−13.5 to 2]; Mann–Whitney *p* = 0.980; data not shown). There were also no significant differences in DBP pre (Group I: −2 [−6.5 to 3.3]; Group C: 1 [−4 to 3]; *p* = 0.750; data not shown) or post-test (Group I: −2 [−6 to 3.3]; Group C: 0 [−4.5 to 5]; *p* = 0.574; see Figure 2C). These results suggest that the intervention promotes a protective effect against the increase in SBP in effort situations.

A heart rate difference was found in Group C in the pre-test measurement (6 [−1 to 12] bpm; Mann–Whitney *p* = 0.030; Wilcoxon *p* < 0.001), which did not occur in Group I (1 [−5 to 7.5], *p* = 0.487). The median of Group C (Figure 2D) in the pre-test was higher in assessment two. An explanation for the effect is that on the day of this assessment in Group C there was warmer weather and that this affected participants’ heart rate. No statistically significant results were found involving post-test heart rate (Group I: 5 [−2.5 to 9.5]; Group C: 6 [−1 to 11]; *p* = 0.599).

Similarly to what was done with blood pressure results, after seeing these results, an analysis for heart rate involving only prehypertensive and hypertensive individuals was conducted. No between-group effects were found in the pre- (Group I: 0 [−5 to 8] bpm; Group C: 6 [−4.5 to 11], Mann–Whitney *p* = 0.444), nor in the post-test (Group I: 6 [−2 to 9.3]; Group C: 0 [−3 to 10.5]; Mann–Whitney *p* = 0.693; data not shown) assessments.

### 3.7 Walk test

A significant Assessment*Group interaction was found (F(1; 86) = 61.33, *p* < 0.001) in the number of meters walked. While Group I had an increase (+54.6 [±57.3] m, *p* < 0.001), Group C showed a decrease (−33.8 [± 50.1], *p* < 0.001). Accordingly, the participants in Group I were able to walk for a longer distance than in Group C when considering only the T1 assessment (Group I: 519 [±51.0]; Group C: 456 [±42.3]; *p* < 0.001; see Figure 2E). This suggests that the intervention increased the walking capacity of the individuals undergoing it.

### 3.8 Flexibility

An Assessment*Group interaction was found in the flexibility assessed by the sit and reach test (F(1; 86) = 5.43, *p* = 0.222; Figure 2F). We noted a difference between T1 and T0 for Group I (+5.12 [±5.60] cm, *p* < 0.001), but not for Group C (−1.82 [±7.48], *p* = 0.260) in the flexibility test. This suggests that there was an increase in the flexibility of participants in Group I, while participants in Group C maintained the same flexibility.

### 3.9 Number of sit-ups

No differences were found between groups in the results involving the number of sit-ups (Group I: 2 [0 to 5], Group C: 0 [0 to 5], Mann–Whitney *p* = 0.176).

### 3.10 Body mass index

No differences were found between groups in the results involving BMI (Group I: 0.379 [0.095 to 0.840], Group C: 0.469 [−0.090 to 1.07], Mann–Whitney *p* = 0.751).

The hypothesis was also raised that changes in BMI would only be seen in participants who had started the intervention with overweight or obesity, i.e., a BMI > 24.9 (67). However, we also did not find differences between T0 and T1 in this population (*n* = 32 in each study group; Group I: 0.395 [0.123 to 0.775], Group C: 0.575 [−0.170 to 0.973], Mann–Whitney *p* = 0.785; data not shown).

### 3.11 Waist-to-height ratio

No differences were found between groups in the results involving the waist-to-height ratio (Group I: −0.003 [± 0.023], Group C: 0.007 [±0.035]). The ANOVA showed no main effect of Group (F(1; 86) = 0.40, *p* = 0.529) or Assessment: (F(1; 86) = 0.39, *p* = 0.534), nor a significant Assessment*Group interaction (F(1; 86) = 2.70, *p* = 0.104; data not shown).

As in the BMI analysis, we hypothesized that changes in the waist-to-height ratio would be observed only in the subgroup of people with BMI > 24.9 (*n* = 32 in each group). However, the ANOVA showed no main effect of Group (F(1; 62) = 0.09, *p* = 0.767) or Assessment: (F(1; 62) = 0.06, *p* = 0.811), nor significant Assessment*Group interaction (F(1; 62) = 1.97, *p* = 0.165; data not shown).

## 4 Qualitative results

A total of 13 participants from Group I, all female, participated in the collective interview.

After familiarization with the data, the transcription was organized in codes. The codes helped to generate 6 themes and their subthemes, as shown in Table 1. Each theme has its meaning described below.

### 4.1 Enhanced well-being

This theme includes 3 subthemes that report how the program has helped improve the participants’ overall well-being. They report feeling better mentally and emotionally, with a decrease in negative emotions like distress, anxiety, and sadness. This suggests the program has effectively boosted their psychological resilience. Participants also experienced an increase in physical vitality and fitness, indicating a positive shift in their energy levels and ability to engage more actively in daily life. Additionally, the program has encouraged the adoption of healthier lifestyle habits, such as increased physical activity and more mindful living. Collectively, these changes demonstrate a comprehensive approach to well-being that integrates mental, emotional, and physical aspects.

*I really liked the program, it was very helpful, not only physically, but especially mentally, socioemotionally, and above all, the practice of meditation. I was a very anxious person, but I was already able to control it. With the help of the project, I managed to control it even more.*

*I feel very well, thank God. After I started, all my muscle pain stopped. I do not feel it anymore. I also had a problem with my knee, it all went away, thank God.*

### 4.2 Relationships and personal growth

This theme reflects the program’s influence on personal development and social interactions, which are pivotal in shaping one’s quality of life. The subthemes explain that Participants experienced positive changes in family dynamics, fostering a more patient and nurturing environment. Additionally, the enhancement of conflict resolution and emotional expression skills implies that the program equipped individuals with tools to navigate and improve their interpersonal relationships. The importance of self-reflection and self-awareness indicates a deepening of self-understanding, which is essential for personal evolution and fulfillment.

*Today I can better handle some feelings that were difficult for me to deal with in the past. In terms of anxiety, today I see it differently… that all of us can go through certain situations and, like me, I can be fine, knowing that I will be that way but, later, everything will improve. So, it’s about trying to see it in a different way.*

*At home, it’s great, I managed to deal with my children, to talk… as I said in the beginning, [in the past] I wanted to hit them right away, I would grab a slipper and say, “look, do not do that,” but now, “come here, let us talk, come here,” and they come…*

### 4.3 Program value and continuity

This theme expresses the appreciation for the program and its perceived benefits. The subthemes highlight a widespread gratitude for the positive personal transformations experienced, as well as an acknowledgment of how these changes benefit the community. The emphasis on the continuation of practices suggests that participants not only value what they have learned but are also keen on integrating these practices into their daily lives for sustained benefit.

*I really liked it [the project]. I just must thank the project you have set up here, which many women take advantage of, and know how to make the most of it, as we did. It was very useful for us.*

*I also intend to continue [the activities]. I will look for a gym if I cannot stay here, of course, right? And I will try to exercise every day, because it really is something indescribable how much more willing you become, you feel happier, more active, right? But it’s very good, you feel better, you understand?*

*It makes us very happy. They [the teachers] made us very happy, always explaining things to us, so it’s very good. We feel very happy about it.*

### 4.4 Self-care and resilience

Self-care and resilience emphasize the program’s role in teaching participants to prioritize their own needs and well-being. By learning and adopting personal care routines, individuals recognize the importance of dedicating time to self-care activities. The subthemes also highlight the acquisition of coping strategies for managing stress and challenging emotions, which is indicative of increased personal resilience. Overcoming interpersonal challenges is a testament to the practical application of these strategies in daily life, particularly in the realm of personal relationships.

*I feel that I learned to take better care of myself, to love myself more. I am someone who thinks a lot about others, so you must learn to take more care of yourself. So, these 3 months for me were wonderful, very good.*

*I was a very anxious person and after I started doing the exercises, I realized that it was… well, 40 min, but it was 40 min that I took for myself. Because I live very much for others, both at work and at home, and it was a moment that I took for myself, I could not think of anything or anyone, just me.*

### 4.5 Community and support

The last theme is about the social component of the program, which stresses the importance of supporting one another and cooperating in a group. It is widely acknowledged that working in teams not only helps people stay on track but also speeds up learning. In other words, creating bonds among communities and fostering mutual aid create an environment that cares about everybody’s welfare collectively rather than just personal development. Thus, this theme represents what it means to share common experiences and draw strength from being part of a community together as subthemes expressed.

*Sports, when practicing in group, makes us feel happier, seeing friends. Now, the project has ended, and we are already missing it, but that is something that, I do not know, is really beyond explanation, the way we interact with people afterward.*

*We miss this little time of ours, as I usually say to the girls… Because I made a lot of friends and there was a day for the group to talk about how your day was… my partner, she looked after the person beside her, she saw that she was stressed, she was worried…*

### 4.6 Findings

The reflexive thematic analysis shows that the Intervention project has demonstrated a profound impact on the participants’ overall well-being, a multifaceted theme that encompasses emotional, mental, and physical health improvements. Participants reported not just a significant alleviation of negative emotional states such as anxiety and sadness, but also a notable enhancement in their physical vitality. These changes demonstrate the program’s holistic approach, which intertwines emotional and physical well-being. This translates into a more energetic and engaged approach to daily activities. The adoption of healthier lifestyle habits further highlights the transformative impact of the program, suggesting that the benefits extend beyond the immediate scope of the training sessions and into the longer-term lifestyle choices of the individuals involved (Table 3).

**Table 3:** Themes and subthemes extracted from the semi-structured interviews via reflexive thematic analysis.

Themes	Subthemes
Enhanced well-being	Improved mental and emotional health. Increased physical vitality and fitness. Adoption of healthier lifestyle habits.
Relationships and personal growth	Positive changes in family dynamics and personal relationships. Enhanced conflict resolution and emotional expression skills. Importance of self-reflection and self-awareness.
Program value and continuity	Gratitude for the program’s positive impact. Acknowledgment of the community benefits. Commitment to ongoing practice and application.
Self-care and resilience	Emphasis on the importance of personal care routines. Coping strategies for managing stress and challenging emotions. Overcoming interpersonal challenges through learned techniques.
Community and support	The supportive dynamic of group involvement. Recognition of group practice efficacy. Community bonds and mutual support encouragement.

## 5 Discussion

The aim of this study was to evaluate the effects of a novel low-cost Socioemotional and Physical Activity Intervention in a vulnerable community during the final period of the COVID-19 global health emergency on the mental and physical health of its residents.

Mental health interventions can be powerful tools for promoting health in individuals and populations. There is, therefore, a need to develop mental health interventions. These should be brief, culturally sensitive and easily taught to both health professionals and non-health-professional participants (68). Given the economic and social consequences of the global crisis due to the COVID-19 pandemic, such interventions have an especially high potential to benefit the populations most affected by this crisis—particularly populations in situations of socioeconomic vulnerability (17). Our study aimed to contribute to this direction, presenting the results from an original low-cost and easily replicable Physical Activity and Socio-Emotional Learning intervention that does not require specific exercise and fitness equipment and relies mostly in the mediation of physical activity and socio-emotional skill instructors—which can be trained members from the same community. The Multidimensional Intervention was carried out in Paraisópolis, a large 58,527 inhabitants vulnerable Brazilian urban community. A quantitative assessment of the effectiveness of the Intervention was made using four validated questionnaires and a physical fitness assessment. A qualitative assessment was also performed, through a reflexive thematic analysis.

We found a reduction in the prevalence of depression symptoms in the participants analyzed, as seen in the results for the depression component of the DASS-21 questionnaire. In the same direction, there was a reduction in the negative score on the PANAS, suggesting a decrease in the occurrence and predominance of negative affections (emotions) in the participants. Taken together, these results indicate a reduction in symptoms correlated with clinical depression in the beneficiary population, suggesting an improvement in mental health. Similar results were found by Kemeny et al., in the original study evaluating Cultivating Emotional Balance, the protocol from which the SE component of the Multidimensional Intervention was based on (21). The development of SE skills in vulnerable communities is relevant considering the high percentage of experiences of adverse childhood experiences experienced by them. Lower self-compassion, greater anxiety symptoms, stress and other indicators of worse socioemotional functioning can be present in these populations (69).

These results are further emphasized by the reflexive thematic analysis. Two of the themes found in this analysis—“Relationships and Personal Growth” and “Community and Support”—are deeply related to social connectedness. A recent scoping review suggests that social connectedness can be considered as a determinant of mental health, protecting adults in the general population from depressive symptoms and disorders (70) and previous studies support that it acts as promoter of mental and physical health (71, 72). Several of the subthemes found (such as “Community bonds and mutual support encouragement” and “Positive changes in family dynamics and personal relationships”) support the notion that not only the simple fact participating in a group for a short time increased the feeling of connectedness, but that the benefits of the Intervention extend beyond the immediate scope of the sessions, increasing participants ability create and maintain meaningful social connections in the future.

As for the physical health of the participants, there was an increase in the number of meters walked in the 6-min test. One interpretation of this result is that the intervention increased the participants’ overall physical endurance, allowing them to walk a greater distance at the same time after the intervention. This result can be promising since the targeted population is highly likely to have suffered from the PA inequalities worsened by the COVID-19 pandemic. For example a study in Thailand identified the most vulnerable groups factors during the Covid-19 epidemic: no income, the unemployed, no access to PA facilities, adults aged 60 + years, low level of education, debilitating chronic disease, no access to PA campaign and low income (73), a group similar to those we may found in this study’s population.

In addition, the results indicate a reduction in systolic blood pressure in hypertensive and pre-hypertensive participants. It is possible to conjecture that the cause of this reduction is because the exercises improved cardiac function, stabilizing blood pressure under high cardiovascular load. This could function as a complement in the treatment of hypertension, a highly prevalent condition. Cardiovascular disease is the leading cause of mortality worldwide and results of the complex interplay of socio-economic, metabolic, behavioral, and environmental factors, and high blood pressure is one of the most important of them (74). Hypertension alone is responsible for 8.5 million deaths worldwide, even though it can be easily detected and treated in primary care facilities (75).

Further, the Intervention was found to increase flexibility in the sit and reach test. The sit and reach test is used for estimating hamstring extensibility (76). Poor hamstring extensibility is highly associated with several health problems, including disk herniation (77) and low back pain (78). It is known that increased flexibility provides a protective effect against osteoarticular injuries, increases range of movement, motor coordination and body awareness, bringing a better quality of life and reducing the likelihood of developing diseases in the future. It is therefore possible that the practice of the Intervention brings these benefits to its practitioners. Flexibility prevents injuries, improves sports performance, and promotes an active lifestyle (79). In a study about the effects of a structured, moderate-intensity physical activity program with 818 participants that included aerobic, resistance, and flexibility training activities compared with a health education program with 817 participants, reduced major mobility disability over 2.6 years among older adults at risk for disability (80).

The subtheme “Increased physical vitality and fitness” that was found in the reflexive thematic analysis corroborates such improvements in physical vitality of the participants. Moreover, this analysis suggests that, in addition to these immediate physical health benefits, the Intervention also improved participants’ health habits; the subthemes “Adoption of healthier lifestyle habits,” “Commitment to ongoing practice and application” and “Emphasis on the importance of personal care routines” support such conclusion.

The physical health results obtained not only show immediate benefits for participants but also suggest lifestyle changes that may continue to improve their lives. It is well established that the practice of regular physical activity leads to large physiological adaptations that improve health, which, for example, decrease the need for medication and promote better health outcomes (81–83). Added to the mental health benefits described above, the ease-of-implementation and relative low cost, the Multidimensional Intervention described here has enormous potential to being scaled up to promote lifestyle changes that improve general health of vulnerable populations. Scaling up interventions, however, has its challenges. Interventions need not only to have proven efficacy and be scalable, but also need partnerships beyond the health sector, institutionalized awareness and support in its implementation and development to scale well (84, 85). Considering that the intervention described here is focused in vulnerable populations, which are mainly attended by public health systems, we believe that the greatest challenge to the scalability of this Intervention would be the enactment of public policies requiring or recommending it (86). However, the fact that it does not require specific exercise and fitness equipment, being designed to allow the training of non-specialists in mental health to deliver it make our Intervention particularly scalable.

Limitations of this study include the fact that we observed a gender imbalance, with more than 96% of participants being women. This was not expected. One possibility is that, although we relied in recruitment channels that we considered strong—namely PECP’s social responsibility initiative and WhatsApp, the most used messaging app in Brazil—it might have led to this imbalance. In this regard, a possible explanation is that women have a closer relationship with PECP because the Institution offers many activities aimed at them. Another is that women tend to dedicate more time to the care of the children and PECP offers a multitude of activities to kids of all ages. A different speculation is that men did not find the activities offered in the intervention did not draw men’s attention—conversations with PECP’s PA teachers reveal that most men in Paraisópolis community only get involved in soccer-related activities (C. A. dos Santos and T. S. Almeida Junior, October 25, 2022, personal communication). Future studies should consider other recruitment strategies as well as evaluating which types of activity might draw the interest of a wider public.

Another limitation is that we tried to carrying out a cross-over study (individuals who were in Group C received the intervention a posteriori and Group I was instructed to continue their activities) but due to (I) delays in the start of the study due to pandemic restrictions before the start of the intervention, (II) the period after T1 included the data collection site’s vacation period and end-of-year festivities, (III) the declaration of the end of the COVID-19 pandemic health emergency period after T1 and (IV) 40% of the participants in Group C dropping out after concluding T1, we considered the subsequent data collection to be highly biased and it was not taken into account in the evaluation. A crossover would have allowed more effective mitigation of confounding variables, such as diet and other life stressors. This limited the present study’s internal validity, and future research should consider ways to address this gap.

Also, it was not possible to control the amount of activity the participants did during the week. Future studies at a minimum track the amount of time participants spent engaging with the videos or in other activities (e.g., by quickly surveying them before every class) to control for this possible source of variability.

Although we used a randomized controlled trial design, expectancy bias was not completely accounted for. At T0 participants were blind in regard to what group they belong to, so it seems reasonable to assume that the T0 data is free from expectancy bias. On the other hand, at T1, the participants did know the group they belonged to, so expectancy bias might have had an influence on it. Future studies should consider using more extensive blinding strategies to further mitigate the effects of expectancy bias.

Despite the fact we found short-term benefits of the Intervention, it is not clear whether it generates benefits in the long-term. Future studies should include at least one additional data collection months of years after the intervention to evaluate the extent to which the Intervention effects persist over time.

Finally, it is important to consider the generalizability of our findings to other populations. The Paraisópolis community, where the study was ran, is a vulnerable urban population, with low average family income (170 to 560 USD) and challenged with unemployment, precarious healthcare access and other challenges (27–29). Although this community has characteristics particular to it, we believe our findings are mostly applicable to favelas and other types of informal settlements (like slums and shantytowns). The generalizability of our results probably decreases as population characteristics get further from the characteristics of the investigated population. It is not possible to rule out, however, that our intervention might be effective in non-vulnerable populations. How different the results of our Intervention are in other populations remains to be investigated. It is also not excluded that the intervention may have positive effects on people diagnosed with mental disorders or other age groups; it should be noted, however, that such cases were not included in the scope of this work.

The main contribution of this study is offering a novel low-cost Multidimensional Intervention that, as we have shown, is effective at promoting mental and physical health in adults from a vulnerable population. The benefits include decreased depressive symptoms, reduced negative moods and emotions, increased social connectedness, better physical endurance and flexibility, improvements in cardiac function on hypertensive and pre-hypertensive people, and the adoption of healthier lifestyle habits. These positive effects were observed within a schedule that required only 1 h of face-to-face time per week for 12 weeks, that does not require specific exercise and fitness equipment, and 12 min of videos sent weekly (2-min physical activity lesson +10-min guided meditation), thus requiring relatively low monetary investment and time. Critically, the intervention was designed to facilitate the training of non-specialists in mental health to deliver it, making it viable as a strategy to promote mental health in times of scarcity of mental health professionals, such as a pandemic.

## Data Availability

The raw data supporting the conclusions of this article will be made available by the authors, without undue reservation.

## Footnotes

^1^Between 900 and 3,000 Brazilian Reais in June 2024.

^2^Marcio Atalla videos is a well-known Physical Educator in Brazil, having hosted and participated in popular Brazilian TV shows, thus the mention to his name was included with the intention of boosting engagement.

^3^50.00 Brazilian Reais.

^4^The food included in the snack offered approximately 300–400 calories and included bread with a slice of cheese or cold cuts, 200 mL of ultra-processed fruit juice and either an apple, a banana, or a small, sweet treat (for example, a cupcake or a chocolate bomb).
