# Supplementary material for: A physical activity and socioemotional intervention for residents of a large vulnerable community in Brazil during the COVID-19 pandemic: a randomized controlled study
Source: Front Public Health. 2025 Mar 14;13:1463401. doi: 10.3389/fpubh.2025.1463401 (PMC11951185; doi:10.3389/fpubh.2025.1463401)
Supplement: Supplementary file 1 [file Table_1.docx]

Supplementary Material

**Table of contents**

[1 Physical Activity component exercise schedule 2](#_Toc190017539)

[1.1 Week 1 2](#_Toc190017540)

[1.2 Week 2 2](#_Toc190017541)

[1.3 Week 3 3](#_Toc190017542)

[1.4 Week 4 3](#_Toc190017543)

[1.5 Week 5 4](#_Toc190017544)

[1.6 Week 6 4](#_Toc190017545)

[1.7 Week 7 5](#_Toc190017546)

[1.8 Week 8 5](#_Toc190017547)

[1.9 Week 9 6](#_Toc190017548)

[1.10 Week 10 6](#_Toc190017549)

[1.11 Week 11 7](#_Toc190017550)

[1.12 Week 12 7](#_Toc190017551)

[2 Socioemotional Learning component lesson plans and contemplative practices scripts 8](#_Toc190017552)

[2.1 Week 1 8](#_Toc190017553)

[2.1.1 Lesson plan 8](#_Toc190017554)

[2.1.2 Contemplative practice script 9](#_Toc190017555)

[2.2 Week 2 10](#_Toc190017556)

[2.2.1 Lesson plan 10](#_Toc190017557)

[2.2.2 Contemplative practice script 10](#_Toc190017558)

[2.3 Week 3 10](#_Toc190017559)

[2.4 Week 4 12](#_Toc190017560)

[2.5 Week 5 13](#_Toc190017561)

[2.6 Week 6 15](#_Toc190017562)

[2.7 Week 7 17](#_Toc190017563)

[2.8 Week 8 17](#_Toc190017564)

[2.9 Week 9 19](#_Toc190017565)

[2.10 Week 10 21](#_Toc190017566)

[2.11 Week 11 23](#_Toc190017567)

[2.12 Week 12 24](#_Toc190017568)

# Physical Activity component exercise schedule

The following sections describe the schedule of exercises week by week. Note that the number of sets and repetitions was not included here because it was adapted based on the fitness levels of the participants present in each session.

## Week 1

Focus: Core Training and Contraction Techniques

**Warm-up:**

- Joint mobility (circles with shoulders, hips, knees, and ankles).
- Light dynamic stretches.

**Exercises:**

- Standing exercises for core and glute activation:
  - Active abdominal contraction (try to pull the belly button towards the spine).
  - Simultaneous contraction of the abdomen and glutes while exhaling.
  - Standing pelvic tilt (slightly push the hips forward while contracting the glutes and abdomen).
  - Light trunk rotation with abdominal contraction.
  - Slow marching in place with active core contraction at each step.

## Week 2

Focus: Breathing Techniques

**Warm-up:**

- Thoracic mobility and diaphragmatic release.
- Guided diaphragmatic breathing.

**Exercises:**

- Types of breathing used in running:
  - 1:1 (inhale for one step, exhale for one step).
  - 2:1 (inhale for two steps, exhale for one).
  - 3:1, 2:3 (variations for fatigue control).
- Application in exercises:
  - Stationary running combined with varied breathing patterns.
  - Light jumps with controlled breathing.
  - Contraction and release movements synchronized with breathing rhythm.

## Week 3

Focus: Joint Movements and Stick Exercises

**Warm-up:**

- Shoulder and hip mobility with a stick.

**Exercises (all exercises using a stick):**

- Chest press.
- Low row.
- Shoulder press.
- Biceps curl.
- Rotator cuff stretch.
- Lunge with stick support.

## Week 4

Focus: Coordination and Rhythm

**Warm-up:**

- Dynamic mobility and small jumps.

**Exercises:**

- Activities with mini hula hoops:
  - Entering and exiting from the front.
  - Entering and exiting from the side.
  - Moving inside the hoop (short and quick steps without stepping out).
  - Coordinated arm movements while moving inside the hoop. Example:
    - Alternating arm circles while moving inside the hoop.
    - One arm rotates forward while the other rotates backward (neuromuscular challenge).
    - Raising one arm while lowering the other while stepping inside the hoop.
    - Quickly switching arm directions while moving laterally inside the hoops.

## Week 5

Focus: Coordination and Rhythm – Part 2

**Warm-up:**

- Faster movement drills.

**Exercises:**

- Combination of hoop entry and exit movements:
  - Open and close feet inside the hoop.
  - Cross-legged steps inside the hoop.
  - Alternating entry and exit with sudden direction changes.
  - Diagonal movement, switching hoops while performing a lateral jump.

## Week 6

Focus: Learning the Squat

**Warm-up:**

- Ankle and hip mobility.

**Exercises:**

- Squat progression:
  - Detailed technique explanation.
  - Half squat.
  - Box squat (for depth reference).
  - Free squat.
  - Overhead squat (with arms raised for better postural control).

## Week 7

Focus: Resistance Band Exercises

**Warm-up:**

- Light movements for muscle activation.

**Exercises (all exercises using resistance bands):**

- Row.
- Shoulder press.
- Bench press.
- Squat.
- Standing hip adduction and abduction.
- Front kicks with the band around the ankle.
- Romanian deadlift (Stiff).

## Week 8

Focus: Exercises with a Tennis Ball

**Warm-up:**

- Ball handling for tactile sensitivity and coordination.

**Exercises:**

- Coordination exercises:
  - Toss the ball up, clap hands, and catch it.
  - Quickly pass the ball from one hand to the other.
  - Squeeze the ball for muscle activation.
  - Drop the ball and catch it before it touches the ground.
  - Pass the ball under the legs.
  - Rotate the ball around the body.
  - Pass the ball over the head, simulating a jumping jack.
  - Bounce the ball on the ground, spin, and try to catch it.

## Week 9

Focus: Circuit Training

**Warm-up:**

- Light combined movements.

**Exercises:**

- Circuit with the following exercises:
  - Box squat.
  - Lunge inside the hoop.
  - Jumping jacks.
  - Stationary running.
  - Lateral dislocation inside the hoop.

## Week 10

Focus: Laterality and Dissociation

**Warm-up:**

- Cross-body movements for neuromuscular activation.

**Exercises:**

- More complex coordination exercises:
  - Lift the right leg while moving the left hand and vice versa.
  - Balance on one foot.
  - Move arms to one side and legs to the other.
  - Right knee touches the left elbow alternately.
  - Quick shifts of foot support while performing arm movements.

## Week 11

Focus: Core Stabilization in Rotations

**Warm-up:**

- Core activation and thoracic mobility.

**Exercises (in pairs):**

- One person holds the ball while the other changes direction.
- The person holding the ball must contract the core to maintain stability.
- Exercises with varying ball positions:
  - In front of the body.
  - Overhead.
  - Standing and kneeling.
  - Applying force to the ball to stimulate isometric resistance (pushing up, down, and sideways).

Applying force in diagonal directions for oblique activation.

## Week 12

Focus: Exercises with Fabrics (Towels, Vests, and Cloths)

**Warm-up:**

- Exploratory movements with the fabric.

**Exercises (all exercises using a cloth):**

- Passing from one side to the other over the cloth.
- Jumping over the cloth.
- Row.
- Shoulder press.
- Squat while holding the cloth overhead.
- Slamming the cloth on the ground (simulating battle ropes).
- Trunk rotation while holding the cloth to activate the core.
- Chest press.

# Socioemotional Learning component lesson plans and contemplative practices scripts

Lesson plans were brief notes that the SE instructor used to introduce the theme of the week to the participants. They were used as a guide, not read to the letter, and always followed by a contemplative practice. The script for these practices were followed to the letter, being read out loud by the SE instructor, with pauses between sentences of variable length based on the practice and the time available.

## Week 1

### Lesson plan

**What I Have to Offer:** Promoting health by caring for emotions

Emotion is a process that begins with stimulation; it involves physiological and behavioral changes. Emotion comes from "to move." It is a movement. It is an expression, including bodily expression—facial expressions.
We are able to interact and feel others because of expressions.
There is an internal programming: a pattern of stimulations in certain parts of the body for each emotion.

From what I see in you, I can decide my next step and plan my next move. This is learning, just as I learn what is happening inside me.

**3 Steps**

1. My medication: situations and discoveries
2. Discussion of a new theme: the way we see life impacts our health
3. Collective meditation

**Meditation**

- Discipline
- It’s not about thinking of nothing or forcing your breathing
- It’s about taming your attention and connecting with your body

**Posture:** Comfortable, still, breathing easily through the nose.
Relax! Relax more!

### Contemplative practice script

To fully enjoy this practice, lie down on your back on the floor, feet hip-width apart, gently roll your shoulders back, open your chest so that your hands naturally face upward.

We begin by bringing to our mind and heart the aspiration, the will to cultivate deep well-being, to calm the body and mind.
Many of the issues that arise during meditation are due to the accumulation of energy in the head area. Tension, pressure, and even headaches… Allow the energy to move down to the torso, the hips, legs, and feet.
Let your attention move to the parts of your body in contact with the floor, to the element of earth—firmness, solidity. Let your awareness rest in this space, quietly present with these sensations.
Trust the support the floor offers to your body.
Now, let your awareness permeate the entire space of the body, like a fragrance filling a room.
Just be present with any sensation that arises in this silent field of perception. And establish your body in a comfortable posture, without unnecessary movement.
Internally adopt a posture of vigilance. Do not allow the initial level of clarity or alertness to diminish throughout this practice.
If the body is relaxed and comfortable, it will be easy to maintain it without any movement until the end of this section… Thus, we establish the body in its natural state: relaxed, still, and vigilant.
Now, we move to a more subtle challenge—establishing the speech in its natural state of silence. Externally you are already silent. But internally, the mental discourse continues. To cultivate this state of effortless silence, we allow the breath to flow in its natural rhythm, without restriction or control.
Let go of even the preference for longer or shorter breaths.

The key here is the exhale. It is the easiest moment to let go, to release tension. Let go of all thoughts, all concerns with each exhalation.
To deepen relaxation, at the very end of the exhale, continue letting go and relaxing... until the next inhale comes almost as a surprise, without effort.
Let go of everything. Hold nothing back. And simply wait for the next inhale to be offered to you.
Whether the breath is deep or shallow, fast or slow, let it be as it needs to be. Let the body breathe, and thus let the breath establish its natural rhythm... Let the body restore its own balance.
And for the short duration of this session, give yourself the freedom to let your mind be at ease... abandoning all fears and expectations about the present and the future. Allow your awareness to rest in stillness, in the present moment—clear, luminous.
Let the luminosity of your own awareness illuminate the sensations throughout the body.
Let the limits of your attention be the space of your body, in the present moment, without wandering into the other physical senses and without being carried away by thoughts.
Let your breath flow. And thus, establish your mind in its natural state—relaxed, still, and clear.
As you feel the sensations in the body, you may notice some tension, especially in the face. We develop many layers of tension. Let them all dissolve, melt away.
Each exhalation is an opportunity to relax. To release.
Try not to let your mind fall asleep, so you can fully enjoy this deep relaxation.
This practice allows the entire body/mind system to restore itself, finding its balance from its root, from its own source.
We now continue in silence.

## Week 2

### Lesson plan

Meditation is also training for daily life. It doesn’t stop at just 10 minutes.
Emotions originate in the brain.
Thoughts come from the way I perceive the world (lens): Genetic, Social, and Personal. Example: the normalization of eating beef versus eating dog meat.
Learning from emotional experiences... Time provides the opportunity but does not guarantee learning.
Mastery of impulses & Refractory period.
“I think too much” vs. Attention: I can focus my awareness.

### Contemplative practice script

Same as week 1.

## Week 3

- - 1. **Lesson plan**

Present and discuss how meditation can influence this process


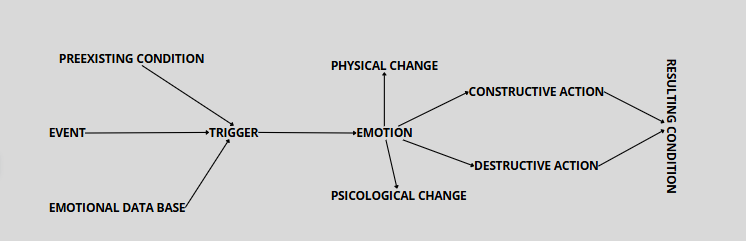


- - 1. **Contemplative practice script**

Let the sound of this bell be a signal for your awareness to return to the body. Here and now.
Establish your most meaningful motivation at this moment and let it give purpose to this practice.
Allow your awareness to descend through the body to the ground, filling the entire space of your body. Maintain awareness in a witnessing mode: clearly attentive but quiet, non-reactive, not trapped by imagination or discursive thoughts. Rest in a state of pure attention.
Establish the body in its natural state: relaxed, still, and vigilant.

Let the body feel at ease. Release all tensions: whether in the shoulders, neck, or back.
Bring attention to the face. Release all tension in the facial muscles.
Relax the eyes, forehead, jaw... let the tongue rest within the mouth... relax the throat... if the mouth remains slightly open, that's okay.
Relax all the muscles of the abdomen so that the breath can flow effortlessly.
And while attentively observing the sensations of breathing, deeply relax with each exhalation. Calm the body; exhalation is a natural occasion for releasing. As the air leaves, relax deeply in both body and mind.
Let go, during these moments, of all concerns about the future or the past.
Maintain your attention on the present moment, on the sensations of the air entering and leaving.

Until now, we have been practicing relaxation, establishing the body, speech, and mind in a state of tranquility. We have cultivated balanced relaxation.
Now we will gently introduce the element of discipline, explicitly cultivating stability, another quality of attention.
To do this, we will be selective, deliberately focusing attention on the movements of the abdomen, rising and falling as the air enters and leaves.
Narrow the focus to the tactile sensations in the abdomen, to the sensations of the air entering and exiting... Keep the belly relaxed.
Even if the breathing is shallow, the abdomen expands as you inhale. If the breath is deeper, the abdomen, diaphragm, and chest expand as you inhale and relax as you exhale.

Remain attentive to the sensations of breathing. Without any preference or interference... the key here is exhalation. Continue relaxing more deeply with each exhalation. Completely release the air, gently letting go of any thought, memory, or mental image. Let the very end of the exhalation be an occasion of total trust. Trust that you can completely release the air until nothing remains, and that there is no need to make any effort to inhale. Let the next inhalation flow in its own time. Be content with what comes. Whether the breath is short or long. Whether the flow is regular or irregular. Trust your body and let it breathe.

You will find a synergy between establishing the body in its natural state and the breath in its natural rhythm. One practice deepens the other.

When the air enters during inhalation, focus clearly on the tactile sensations of the belly expanding. Intensify, focus your attention when the air enters... Then, when the air begins to leave, relax. Let go.
Gently sustain the flow of attention, engaged with the sensations of breathing, and relax during the exhalation.

Focus more attention on inhalation and completely relax during exhalation.
Focusing with more attention is a natural antidote to the tendency of attention deficit—lethargy, drowsiness, torpor... And completely relaxing during exhalation is a natural antidote to the hyperactivity of attention, agitation, distraction... Release the energy associated with tension.

To the best of your ability, let your attention remain engaged with the sensations of the abdomen rising and falling throughout the course of breathing. Like a rider on a horse.

Stay connected to the entire flow of the breath, attentive to breathing as a stream.
This is a continuous task. By filling your attention with the sensations of breathing, there is no time for the mind to wander among memories, fantasies, and errant thoughts.
Engage your attention 100% of the time with the sensations of breathing.

Especially with each exhalation, simply let go of any image, thought, or memory that arises in your mind, without a second thought, without linking ideas.
Let them go and gently bring your attention back to the tactile sensations of the abdomen rising and falling during breathing.
And if your awareness loses interest and becomes drowsy, renew your interest in the practice. Reestablish the clarity of attention and return to focus.

Choose, at each moment, peace and stillness over stimuli. The field of tactile sensations is silent by nature. The mind naturally calms down.

We now continue in silence.

And now, in the final moments of this practice, let your awareness simply rest in its own space, illuminating itself.

## Week 4

- - 1. **Lesson plan**

**Theme:** Being prevented from achieving something important.
**Message:** “Get out of my way.”
**Effects:** Anger controls, punishes, and retaliates.
**Dangerous effects:** Anger breeds more anger.

- Injustice
- Stupidity
- Disrespect
- Frustration over unmet desires
- Being misunderstood
- Betrayal, abandonment
- Being falsely accused
- Breaking a cultural rule (being invaded)
- Anger toward others

**Anger is rarely alone…**

- Fear of being hurt by the target of anger, hurting the target, or losing control.
- Aversion/Disgust toward the target or oneself.
- Shame or Guilt for feeling or expressing anger.

**Why are many episodes of anger directed at people we love?**

- **Proximity:** They can hurt and disappoint us more than others.
- **Romantic love:** We may fantasize qualities in the loved one that don’t exist and become disappointed.
- **Safety:** It may feel safer to show anger toward people we’re intimate with than toward strangers.

**Anger can be a problem when:**

- It occurs too frequently.
- It is too intense or too weak.
- It leads to violence.
- It interferes with relationships with others.
  - 1. **Contemplative practice script**

Same as week 3

## Week 5

- - 1. **Lesson plan**

**Previous Lesson (Anger):**Pauses throughout the day: breathing, noticing tensions, and cultivating presence (escaping the future—anxiety—and the past—regrets). *Image of deep breathing.*

**Joy**

- Motivates our lives.
- From an evolutionary perspective, it encourages participation in activities essential for the survival of the species.

**Constructive Joy:**

- Potential to promote connection, collaboration, and care for ourselves and others.
- On a deeper level, it can lead to the pursuit of happiness, the cultivation of wisdom, and compassion.

**Destructive Joy:**

- Pleasurable experiences that antagonize our genuine happiness or that of other beings, including animals.
- Pleasures that disrupt our balance due to fixation or obsession.

**Universal Experiences of Joy:**

- Reuniting with loved ones.
- Being in the presence of the beloved.
- The birth of a wanted child.
- Loving and consensual sexual relations.
  - 1. **Contemplative practice script**

We begin the practice by setting the intention to cultivate deep well-being and to use this practice as support on your path toward genuine happiness, for your own benefit and for the benefit of those around you.

Establish your body, speech, and mind in their natural states: tranquility, calmness, and alertness. With an expression of care and loving-kindness toward yourself.

The quality of empathetic joy, in its deepest expression, means celebrating happy moments of connection, simple joys, and also the virtues and skills we can offer in service to others.

Start by remembering a joyful moment when you were in the presence of someone dear to you, or when you were alone enjoying the comfort of your home, the beauty, or the tranquility of a place. Let this moment infuse your here and now.

Feel yourself in that situation again and see if you can access that pleasure once more. Rejoice in having had this opportunity and in knowing that other joyful situations will surely arise in your life. Rejoice in this!

Now bring to mind a situation where you expressed a skill you possess: creativity, competence, responsiveness, intelligence—any skill, simple or sophisticated.

Recognize that, in that moment, you did your best.

Now recall a situation where you were able to express a virtue—generosity, compassion, altruism, kindness, gentleness—any situation. One or more situations.

Acknowledge that you are capable of expressing this virtue and many others. Rejoice in this!

When we recognize our skills and virtues, we feel that they are available to us. And when we remember the many people who helped us develop these virtues or skills, gratitude saves us from pride.

I now invite you to remember the various people who have impacted your life, taught you, offered to help, served as models, or inspired you throughout your life. See if your heart can open and express gratitude.

Prepare to close the practice.

## Week 6

- - 1. **Lesson plan**

**Theme:** Real, imagined, expected, anticipated, or misinterpreted threats
**Message:** “Help me”; it can range from mild concern to panic.
**Reactions:** Fear leads to freezing (in most primates) or fleeing if the threat persists. Fear and anger can easily alternate, and fighting may be motivated by anger mixed with fear.

**Triggers**

- Possibility of physical or emotional pain
- Loss of bodily support
- Object moving rapidly in the visual field
- Reptilian shapes – these can be learned from a single exposure associated with physical pain; fear of knives and firearms requires longer training
- Animals are more predisposed to fear than humans. Example: a mouse fears a cat without needing to learn it
- Most of our fears are learned
  - 1. **Contemplative practice script**

We begin this practice by bringing the body into a state of comfort, relaxation, and ease...

Establish the body in its natural state of relaxation, stillness, and vigilance.

Let the body breathe without any obstruction, without any preference, and without effort.

Now we move into a discursive practice that will allow us to cultivate qualities of the heart, especially loving kindness and compassion.
At this moment, we will use imagination.

Invite a loved one to join this practice with you, mentally...

Bring the clearest image possible of this person, as if they were sitting right in front of you.

With your eyes closed, imagine that this person sitting before you in your mind’s space. Imagine that this person has been disappointed by someone, suffered a loss, and became sad, exactly as you have.

Imagine that this person wishes to be free from suffering, distress, anxiety, and loneliness, just as you do.

Feel the connection between you and this person in front of you, and with each exhalation, send them the following wish, the following aspiration: “May you be free from suffering and all the causes of suffering.”

Now, consider that this person sitting in front of you in your mind’s space has also rejoiced for many reasons, has felt loved, and has loved. This person has felt happiness simply by giving their best. Notice how you feel imagining that this person has had many moments of joy, of genuine happiness. Notice how you feel.

Now, with each exhalation, send them the following aspiration: “May you cultivate genuine happiness, independent of any external circumstances. May you cultivate fearlessness, knowing that you already have everything you truly need.”

Notice how you feel as you give these wishes, this aspiration, to this person...

Now, bid farewell to this person and see who comes to sit in front of you, a new person.
See if it’s possible to wish for this person sitting in front of you in your mind’s space to be free from suffering and all the causes of suffering.

See if it’s possible to wish for them to find happiness and cultivate all the causes of happiness...

See if it’s possible to make this wish for happiness and liberation from suffering for any person who comes to sit in front of you.

Now, imagine that you yourself are sitting in front of you, in your mind’s space...

Make the same aspirations, the same wishes for yourself...

May I be genuinely happy...
May I cultivate all the causes of happiness...
May I overcome all causes of suffering, all suffering... without fear...
And may I open my heart to all beings...
Give my best to each one of them... whoever they may be...

And now, in the final moments of this practice, simply rest.
Let go of the aspirations, the wishes, and rest with an open heart... in peace.

## Week 7

- - 1. **Lesson plan**

...what to do with so many meditations: Practice for 1 week to begin. Then choose, knowing how your body, mind, and heart respond to each one.

**Hedonic Happiness or Hedonism**Hedonic happiness comes from the presence of positive emotional states and the absence of pain. It dependent on the outcome. From the outside in. I consume from the world. The fulfillment of our basic needs is included here.

**Eudaimonic Happiness or Eudaimonia**It is based on who we are, not what we obtain. It’s not dependent on external stimuli and does not diminish as it’s more experienced. It is cultivated internally. Life purpose. I give to the world.
Often translated as genuine happiness or human flourishing, it is a way of life, a good life, the satisfaction and sense of accomplishment that derives from the qualities we bring to the world. Quality & Longevity.

- - 1. **Contemplative practice script**

Same as week 7.

## Week 8

- - 1. **Lesson plan**

**“An inattentive mind is an unhappy mind.”**

**Fear and Anxiety**

**Common Mental States**

- Desire/Attachment/Longing
  (fear of losing, anxiety, or effort to obtain something)

**Desire / Attachment (Longing)**

- The desire to sustain pleasurable sensations by clinging to something or someone as if they were a true source of happiness.
- Implicit dissatisfaction from always wanting more, making us susceptible to different emotional experiences when we don’t get what we want and receive what we don’t want – frustration, anxiety, disappointment, hostility.
- In the absence of desire/attachment, there is contentment. In a state of contentment, the mind easily rests with a quality of stillness.

**The Body Under Fear:**

- Hormones flood the body
- Heart accelerates
- Blood pressure rises
- Oxygen is directed to activate important muscles
- Sweat is released to cool the muscles
- Non-essential systems, like digestion, are shut down
- Hormones cause your attention to focus on the threat and exclude everything else… The cause? An immediate threat.

**The Body Under Anxiety:**

- ... The cause? Your thoughts
  - 1. **Contemplative practice script**

We begin this practice by bringing comfort to the body... Relax all tensions, allowing each exhalation to be a natural opportunity to let go...

Become more deeply familiar with this experience... of releasing... of allowing your body to feel at ease and peaceful, finding a natural state of stillness and quietness... one you can maintain effortlessly...

Maintain an internal posture of vigilance... this way, we cultivate a balance between sustaining deep relaxation while remaining simultaneously alert... with an open chest, a relaxed diaphragm, and a loose and relaxed abdomen, allowing the breath to flow naturally...

Offer yourself this opportunity to, just for a few moments, let go of all concerns about the past and the future... all fears and expectations...

Let your mind feel at ease, peaceful, quiet... in its natural clarity... illuminating the entire space of your body and all the sensations arising within this space...

Now we will gently introduce the element of discipline, explicitly cultivating stability, another quality of attention.

To do this, we will be selective, intentionally focusing attention on the movements of the abdomen, rising and falling as the air enters and leaves. This time, in a special way.

Narrow the focus to the tactile sensations in the abdomen, to the sensations of the air entering and leaving... Keep your belly relaxed.

Now, we will breathe in a special way. I invite you to take deeper breaths... always inhaling through the nose, count to 4 as you inhale. Feel the air filling your lungs and your abdomen rising. Hold the air in your lungs for 2 seconds and exhale, counting again to 4.

Even if the breathing is shallow, the abdomen expands as you inhale.

Try to maintain this rhythm of deep breathing and return your attention to the tactile sensations in the abdomen as soon as possible. The goal is to connect with the body and not just count.

... Remember to take the opportunity to relax more and more with each exhalation. ... And take advantage of the inhalation to reawaken your state of alertness and attention. ... If your mind wanders, it's okay. Celebrate noticing it. Simply return your attention to the breath.

Now, a new invitation: return your breathing to its natural flow. No counting. No preference. No interference. Remain simply attentive to the sensations of breathing in the abdomen.

... Whether the breath is short or long. Whether the flow is regular or irregular. Trust your body and let it breathe. ... Completely release the air, gently letting go of any thought, memory, or mental image. ... Let the very end of the exhalation be an occasion for total trust. Trust that you can release all the air until nothing remains, and that there is no need to make any effort to inhale. Let the next inhalation flow in its own time. Be content with what comes.

Prepare to close the practice.

## Week 9

- - 1. **Lesson plan**

**Theme:** Allowing connection in the face of loss
**Message:** I need comfort

Sadness is one of the most enduring emotions, almost like a mood.
Sadness and Agony are two sides of the same emotion; sadness is the more resigned side; agony is the side that protests and tries to cope more actively.
Grief is a multifaceted reaction to loss.
Sadness is an essential characteristic, but it is not the only one. Anger and guilt can also be present.

**Triggers**

- Many types of loss
- Rejection by a friend
- Loss of a job
- Deterioration of the body through illness or aging
- Loss of a situation where one is admired or praised

**Constructive**

- Induces comfort; when people feel discouraged, they are generally seeking comfort, not a solution.
- Creates connection with others who have experienced similar losses.
- When we open ourselves to experience sadness, we may develop, over time, the ability to accept the fragile and temporary nature of our lives; we are reminded to live fully every day and moment.

**Destructive**

- When it distorts our view and leads to despair
- Vulnerability to losses may lead us to avoid commitment with others
  - 1. **Contemplative practice script**

This time, I invite you to joyfully let go of all the content of your mind and enjoy the stillness of your body and consciousness.

It is in this stillness that we will find the deepest well-being, the most authentic joy. This is where we must rest, free and unburdened.

With this joy, gently allow your body, your breath, and your mind to settle into their natural states: tranquility, stillness, immobility, and relaxation.

Relax all the tensions. All the contractions throughout the body: from the forehead down to the feet. Just witness your body breathing.

Now, to support you in this practice, invite a person whose mere presence fills your heart with joy. It could be someone very dear to you, or someone cheerful, full of life, always offering a smile... How do you feel in the presence of this person?

Feel how your body reacts, how your heart feels. Feel the joy you experience when sharing in their joy.

Then thank them and say goodbye.

Now invite a person you consider very courageous, someone who faces life’s challenges with grace, wisdom, and firmness. Remember situations where they demonstrated this virtue. Notice if witnessing this quality brings joy to your heart. Observe whether you feel joy and happiness seeing this person express this quality, and perhaps inspiring others.

Then thank them and say goodbye.

Now invite people who manifest other qualities you admire: compassion, wisdom, generosity, kindness, any virtue that is important to you. In the same way, let the expression of these virtues fill your heart with joy.

Invite as many people as you wish.

Thank them and say goodbye to each one.

Now reflect on the kindness that repeatedly manifests in the world, everywhere, in various situations. The kindness of mothers, fathers, great leaders, very poor people, very rich people, animals, communities, professionals... There are so many expressions of kindness, compassion, altruism, generosity... Qualities that should be celebrated, to inspire us, to inspire everyone.

And in the last moments of this practice, just rest, present, with an open heart.

## Week 10

- - 1. **Lesson plan**
- Highlight the relationship between meditation (attention training) and our connection with our more subtle emotions.
- Suggest that participants list 10 good things that have happened to them in the past 3 hours:
  - 1st mentally
  - 2nd sharing some with the group
- Emphasize the importance of connecting with the good things in life (both hedonic and eudaimonic) to cultivate emotional balance and hope in difficult times.
  - 1. **Contemplative practice script**

We begin this practice by bringing into our mind and heart our highest desire, our aspiration, which includes the deepest cultivation of the qualities of the heart.

Bring your attention to your body, and as an expression of self-compassion, release all tension, all rigidity, and gradually bring your body to a state of comfort, at ease.

Let your breath flow naturally, without effort or obstruction.

And at least for the duration of this session, again as an expression of self-compassion, allow yourself to rest, apart from all worries about the past and the future, from all the noise in the mind. Rest in the silence of the sensations of the breath.

It is said that the qualities of love, compassion, joy, and equanimity are the natural expression of a healthy mind, but that we can and must cultivate them until they truly become spontaneous.

And it is always best to begin this cultivation from the simplest to the more complex. We begin by mentally inviting to help us in this practice someone who easily awakens our love, our good-will. Feel this person’s presence in front of you.

Recognize that, just like you, they wish to be happy and not suffer. And they will certainly face challenges, more or less difficult, but the nature of our experience is this, filled with pleasant moments and others not so much. However, this dear person, like you and like anyone else, has potentially all the resources they need to deal with any challenge.

Remember that love, when it encounters suffering, becomes compassion. They are two sides of the same coin.

Now, from this place that empowers, that recognizes the abilities of the being in front of you, and also understands that challenges are inevitable, make aspirations like:

“May you transcend all suffering. May you recognize and abandon all the causes of suffering. May you cultivate all the resources to deal with the challenges that arise in your life.”

Let this desire for this dear being to be free of suffering transform into a readiness to do whatever is possible to help them, even if not now, even if you do not yet know how.

Let this desire and readiness fill your heart.

When you feel ready, say goodbye to this dear person, thanking them for supporting your practice, and wait for whoever may knock at the door of your mind. Invite this next person to sit in front of you and repeat the same practice you did with the dear person. Whoever it may be, this person too certainly wishes to be happy and not to suffer, just like you and just like the dear person. Repeat the same practice.

“May you transcend all suffering. May you recognize and abandon all the causes of suffering. May you cultivate all the resources to deal with the challenges that arise in your life.”

When you feel ready, say goodbye again and welcome the next visitor. See if it is possible to repeat the same practice with these people.

Now, say goodbye to this last person who participated in your practice.

Return your attention to yourself. Rest for a few moments in touch with the qualities of love and compassion present in your heart.

## Week 11

- - 1. **Lesson plan**

Many emotional pains occur (and persist!) when I distance myself from the "obvious." When I lose sight of aspects that are part of the human experience.

**Impermanence – A Natural Condition: Everything Ends (Changes)**

- Do I understand that changes are inevitable?
- Do I pray for things to never change?
- Do I notice that people change?

**Equanimity – A Natural Condition: There Are Differences**Another factor that distances us from balance and genuine happiness is judgments and categorizations:

- Like: Attachment
- Dislike: Anger (Aversion to the different)
- Indifference: Coldness

We even do this with our emotions! For example: "This pain of mine is not that important."
Consequence: I don't validate it, and I don't take care of it.

**Equanimity**Sustaining an impartial view with respect to all beings, without categorizing them.

- - 1. **Contemplative practice script**

Same as week 10

## Week 12

- - 1. **Lesson plan**

Propose a brief discussion circle about the journey.
Highlight that participants now possess extensive knowledge and can continue practicing.

- - 1. **Contemplative practice script**

We begin this practice by bringing comfort to the body... Relax all tensions, allowing each exhalation to be a natural opportunity to let go...

Become more deeply familiar with this experience... of releasing... of allowing your body to feel at ease and peaceful, finding a natural state of stillness and quietness... one you can maintain effortlessly...

Maintain an internal posture of vigilance... this way, we cultivate a balance between sustaining deep relaxation while remaining simultaneously alert... with an open chest, a relaxed diaphragm, and a loose and relaxed abdomen, allowing the breath to flow naturally.

Let your body breathe without any interference... without control or effort...

Offer yourself this opportunity to, just for a few moments, let go of all concerns about the past and the future... all fears and expectations...

Let your mind feel at ease, peaceful, quiet... in its natural clarity... illuminating the entire space of your body and all the sensations arising within this space...

Now, we move into a moment of celebration and gratitude. For this, I invite you to say the following phrases to yourself, while also feeling how they resonate within your body and heart:

- I celebrate the opportunity given to me to participate in this project.
- I celebrate my dedication to the extent that I could.
- I celebrate having received instructions, knowledge, and support on this journey.
- I celebrate being here... today... in the only moment that exists. Here and now.

For a few moments, observe how your heart and body respond to this celebration.

Now, I invite you to sincerely thank yourself for everything that has happened on this journey...

Now, I invite you to say to yourself, with deep sincerity:

- I thank myself for having accepted this opportunity.
- I thank myself for being open to the new.
- I thank myself for doing what I could.
- I thank myself for making the effort I desired.

And now, feeling these qualities of the heart, we will remain in silence for a few moments.
